# A novel program of cadaver surgical training for young surgeons at the Clinical Anatomy Laboratory Nagoya (CALNA)

**DOI:** 10.1007/s11748-025-02157-8

**Published:** 2025-05-16

**Authors:** Shota Nakamura, Harushi Ueno, Yoshito Imamura, Shoji Okado, Yuji Nomata, Hirofumi Takenaka, Hiroki Watanabe, Yuta Kawasumi, Yuka Kadomatsu, Taketo Kato, Tetsuya Mizuno, Toyofumi Fengshi Chen-Yoshikawa

**Affiliations:** Department of Thoracic Surgery, Nagoya University Graduate School of Medicine, 65 Tsurumai-cho, Showa-ku, Nagoya, 466-8550 Japan

**Keywords:** Cadaver surgical training, Surgical education, Off-the-job training, General thoracic surgery

## Abstract

**Purpose:**

This study aimed to evaluate the effectiveness of a cadaver surgical training program at the Clinical Anatomy Laboratory Nagoya (CALNA), focusing on its impact on procedural skills, confidence, and anatomical understanding among young and mid-career thoracic surgeons.

**Methods:**

From 2016 to 2024, 13 cadaver surgical training sessions were conducted, divided into basic, advanced, and specialized courses. The program included hands-on practice using cadavers preserved with Thiel or hypertonic saline methods. The surveys were administered post-training to assess confidence, satisfaction, and practical applicability. Statistical analysis was performed on the survey results.

**Results:**

A total of 100 participants attended the training sessions (mean: 12.5/session). The survey responses indicated that 92% of participants rated the training content as “good” or “excellent,” and 88% found the training “applicable” or “highly applicable” to clinical practice. Reflective discussions following each session facilitated iterative program refinement. The key improvements included enhanced surgical instrument availability and optimized trainee-to-instructor ratios.

**Conclusions:**

Our cadaver surgical training program was shown to significantly enhance surgical skills, boost confidence, and deepen thoracic anatomical understanding, demonstrating its value in advancing thoracic surgical education. Further development of standardized programs across institutions is needed to enable novice surgeons to acquire advanced skills efficiently.

**Supplementary Information:**

The online version contains supplementary material available at 10.1007/s11748-025-02157-8.

## Introduction

Surgeons have predominantly relied on on-the-job training to develop their surgical skills, where proficiency is traditionally gained through practice during actual surgeries. However, this approach is increasingly viewed as less favorable due to ethical concerns and patient safety considerations, leading to a growing demand for off-the-job training that allows surgeons to refine their skills in simulated environments before performing procedures on patients. Off-the-job training includes various methods, such as simulation-based training, hands-on practice using artificial organ models or resected animal organs, training with live large animals, and cadaver surgical training [1–3]. Additionally, nontechnical training programs, such as case studies, scenario-based training, and mental conditioning for surgeons, are becoming more sophisticated and integral to comprehensive surgical education [4, 5].

Animal-based training, particularly using live pigs, offers valuable practical experience due to the realism it provides. However, this approach has become less acceptable because of anatomical differences from humans and growing concerns for animal welfare [6]. Conversely, human-like tissue conditions can be maintained due to advancements in cadaver preservation techniques, such as the use of specialized embalming solutions, making cadaver surgical training an increasingly established component of off-the-job training [3, 7].

We conducted 13 cadaver surgical training sessions to enhance the surgical skills of young and mid-career surgeons and provide training in complex surgical techniques. Consequently, we developed a program that enables surgeons to safely perform procedures, such as thoracoscopic surgery, in real clinical practice after training [8]. This study aimed to evaluate the effectiveness of our cadaver surgical training program, focusing on its impact on participants’ procedural skills, confidence, and anatomical understanding through a survey of participants. We hypothesized that this structured training significantly improves accuracy, independence, and safety of thoracic procedures, thereby preparing surgeons for real-world clinical applications.

## Materials and methods

### Ethical consideration

Cadaver surgical training was performed in the Clinical Anatomy Laboratory Nagoya (CALNA) at the Nagoya University Graduate School of Medicine, an on-campus facility that adheres to the guidelines for cadaver dissection in clinical medical education and research [9]. The cadavers were fixed in a Thiel solution containing formalin and propylene glycol (Thiel’s fixation solution, A.S. Chemical, Osaka, Japan) or the hypertonic saline method [10, 11]. We conducted this research in the clinical anatomy laboratory at the authors’ institution, and the institution’s Ethics Committee approved this study (no. 2017-0487).

### Training program

The cadaver surgical training was conducted to enhance thoracic surgical skills. The training sessions were organized biannually, with each session lasting one full day, divided into morning and afternoon training periods. The participants were young surgeons, mid-career thoracic surgeons, and supervisors from various institutions. Before the training day, the participants were encouraged to review the training goals and procedural steps with the supervisors to optimize preparedness. In each session, they were grouped at specific tables dedicated to a particular surgical skill or procedure, under the guidance of experienced instructors. The trainees can practice all procedures using the same surgical instruments used in actual surgeries, such as electrocautery devices, energy devices, and forceps designed for video-assisted thoracic surgery (VATS). All training courses are designed to last two hours. Each table is generally composed of 2–3 trainees, 2 supervising physicians, and approximately 2 supporting physicians, though these numbers may vary depending on participant availability.

The cadaver training course was divided into several skill levels to address a range of thoracic surgical procedures, from foundational techniques to complex, specialized skills. The course included basic and advanced skills courses, a VATS course, and lung procurement training.

Basic skills course: the trainees learned the fundamentals of thoracic surgery, including standard thoracotomy approaches, pulmonary artery dissection, and anatomical lobectomy. To understand the chest anatomy more thoroughly, additional training was provided in median sternotomy.

Advanced skills course: this session covered less frequent but essential approaches, such as the clamshell incision, hemi-clamshell, and the transmanubrial osteomuscular sparing approach (TMA), which allowed the participants to practice complex techniques in thoracic access (Supplemental Video 1).

VATS training course: the participants performed VATS lobectomy by using realistic VATS instruments and energy devices, which simulated the operational environment of a live surgery (Supplemental Video 2).

Lung procurement training: the trainees performed sequential cardiac and lung procurement with cardiac surgeons, focusing on the anatomical landmarks and procedural precision necessary for organ retrieval. Key skills included dissecting the left atrium precisely, understanding pulmonary perfusion drainage, and mastering essential timing and handling techniques crucial in lung and heart transplantation.

After each session, the participants engaged in a reflective discussion, where they shared their achievements, identified areas for personal improvement, and discussed challenges during procedures, which helped refine the program over time. Recurring feedback informed adjustments to the training structure and content to better suit the needs of young thoracic surgeons.

### Evaluation and feedback

The effectiveness of the cadaver surgical training program was assessed through a post-training self-evaluation survey, skill assessment, and reflection process. The participants were asked to complete a structured questionnaire before and after each training session to evaluate their confidence in performing specific surgical procedures, overall satisfaction, and areas of improvement. They were surveyed using a structured questionnaire. (Table 1). Open-ended feedback section: quantitative feedback was collected on various aspects of the training, including the adequacy of training duration, participant-to-instructor ratio, and the quality of the provided equipment. Additionally, the participants were encouraged to provide qualitative feedback, highlighting aspects they found beneficial, areas needing improvement, and suggestions for future training sessions. During the 1st to 4th sessions, the training program was in its startup phase and was conducted through a process of trial and error without a fully established curriculum. From the 5th session onward, the training program and its content were improved. Accordingly, we were able to conduct systematic surveys starting from the 5th session.

**Table 1:** Structured questionnaire for cadaver surgical training evaluation

Question	Scale
Number of trainees	1 (Too few), 2 (Slightly few), 3 (Appropriate), 4 (Slightly many), 5 (Too many)
Number of instructors	1 (Too few), 2 (Slightly few), 3 (Appropriate), 4 (Slightly many), 5 (Too many)
Start time of the training	1 (Too late), 2 (Slightly late), 3 (Appropriate), 4 (Slightly early), 5 (Too early)
End time of the training	1 (Too late), 2 (Slightly late), 3 (Appropriate), 4 (Slightly early), 5 (Too early)
Duration of the training	1 (Too short), 2 (Slightly short), 3 (Appropriate), 4 (Slightly long), 5 (Too long)
Quality of the training content	1 (Poor), 2 (Somewhat poor), 3 (Average), 4 (Good), 5 (Excellent)
Overall satisfaction	1 (Very dissatisfied), 2 (Dissatisfied), 3 (Neutral), 4 (Satisfied), 5 (Very satisfied)
Was the training applicable to practical use the next day?	1 (Not applicable at all), 2 (Not applicable), 3 (Neutral), 4 (Applicable), 5 (Highly applicable)
Open-ended feedback section	Participants provided additional qualitative feedback on various aspects of the training

### Statistical analyses

We used Microsoft Excel (Microsoft Corporation, Redmond, WA, USA) for statistical analyses to compile the survey responses and generate graphical representations. The descriptive statistics were calculated for each survey question, and bar charts were created to visualize the distribution of responses.

## Results

### Participant demographics and session overview

**Fig. 1:**
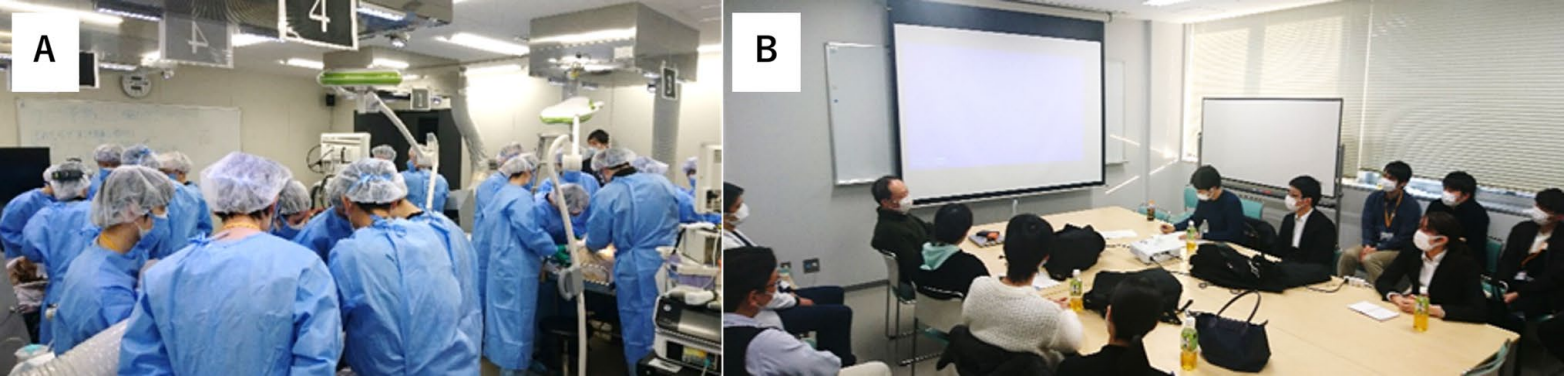
**A** A cadaver surgical training conducted at our department that included young surgeons, mid-career thoracic surgeons, and supervisors. Thirteen cadaver surgical training sessions were conducted from 2016 to 2024 at CALNA. **B** Reflective discussions, an integral part of the training, are conducted after each session. All the participants shared their achievements, identified areas for personal improvement, and discussed the challenges during the procedures. This process helped refine the program over time, with recurring feedback informing adjustments to the training structure and content to better suit the needs of young thoracic surgeons

We conducted 13 cadaver surgical training sessions from 2016 to 2024 at CALNA (Fig. 1A). In the 5th to 12th sessions, 100 individuals participated overall (mean: 12.5 participants per session). The participants included young surgeons (*n* = 35), mid-career thoracic surgeons (*n* = 38), and supervisors (*n* = 27). Each session followed a structured schedule, including morning and afternoon training periods, and concluded with reflective discussions (Fig. 1B).

### Survey responses and feedback

**Fig. 2:**
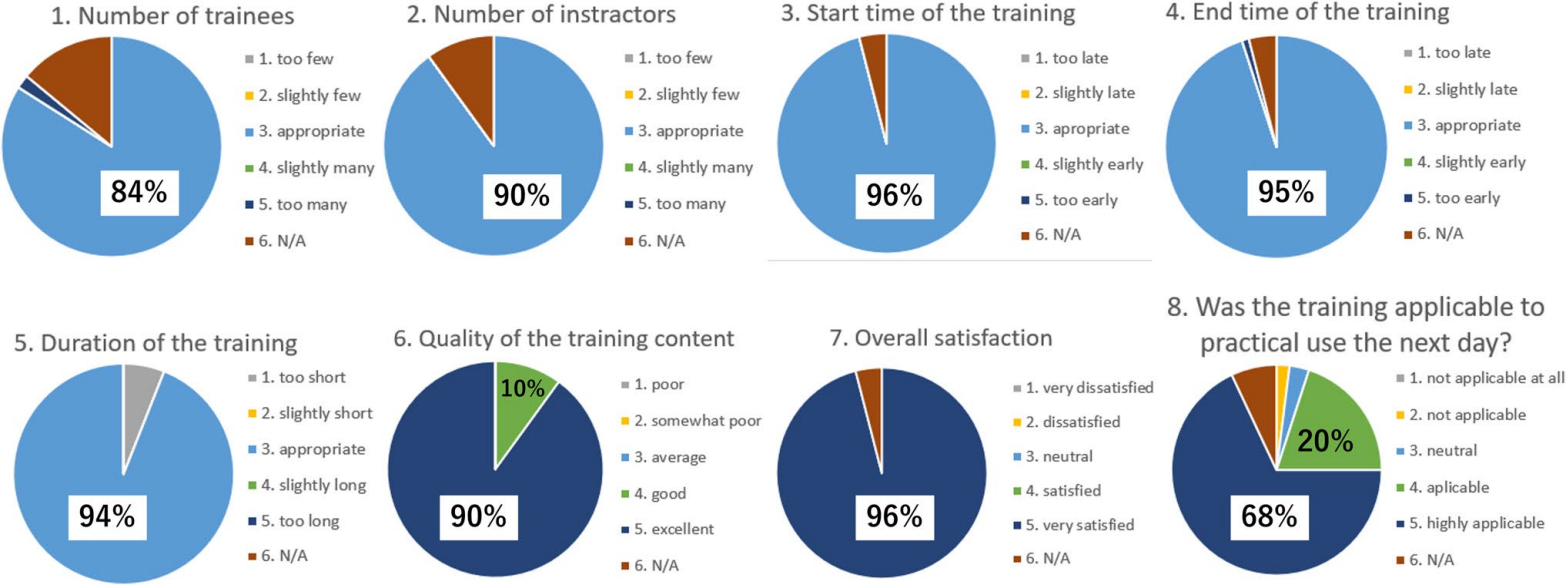
Summary of the results of the survey. *N/A; not applicable

The survey response rate was 100%, with all participants completing the post-training evaluation questionnaire (Fig. 2). Across all sessions, most participants (85%) rated the number of trainees as “appropriate” (score 3). Similarly, 90% of participants considered the instructor-to-trainee ratio as “appropriate” (score 3). Nearly all participants (95%) agreed that the start time was “appropriate” (score 3). Approximately 85% of participants found the end time as “appropriate” (score 3), with minor feedback suggesting slight adjustments for session efficiency. Most participants (86%) indicated that the duration of the training was “appropriate” (score 3). A significant proportion of participants (92%) rated the training content as “good” or “excellent” (scores 4 or 5), highlighting the hands-on experience with advanced surgical techniques as particularly beneficial. All participants reported being either “satisfied” or “very satisfied” (scores 4 or 5) overall with the training program. Approximately 88% of participants considered the training as “applicable” or “highly applicable” (scores 4 or 5) to their clinical practice.

### Qualitative feedback

Participants provided open-ended feedback, highlighting several positive aspects of the training. These included realistic surgical conditions using cadaver models with high anatomical fidelity and the opportunity to practice rarely performed but critical surgical techniques, such as lung procurement and advanced thoracic approaches. In addition, participants suggested several areas for improvement, some of which have already been addressed:

Insufficient surgical instruments: essential instruments were purchased. Additionally, we received donations of surgical instruments that were scheduled for disposal from nearby institutions, allowing for their reuse.

Limited training time due to several trainees per table: we aimed to limit the number of trainees to two per table whenever possible. However, due to the limited number of cadavers, there were instances where three trainees per table were unavoidable.

Provision of preparatory learning materials (texts and videos): this remains an ongoing task. Addressing this issue would require a stable supply of cadavers, the development of a more systematic program, and secured funding.

Proposal to connect the cadaver to a ventilator to assess air leaks: although this suggestion was raised, limitations in budget and concerns regarding exhaled air safety have prevented its implementation. We recognize this as an important issue to be addressed in the future.

These refinements have been incorporated progressively to optimize the effectiveness and accessibility of the training program while maintaining a high level of realism and educational value.

### Program refinement

Considering the recurring feedback from participants, we implemented several improvements during the 11th to 13th cadaver surgical training sessions to enhance the program’s effectiveness and participant experience. Specific adjustments included expanding the variety and availability of surgical instruments to accommodate diverse training needs and reducing the number of trainees per table to increase individual hands-on time and ensure a more focused learning experience. Additionally, we optimized participant-to-instructor ratios to provide more personalized guidance during training and introduced additional equipment tailored for VATS procedures, enabling a more realistic simulation of thoracoscopic surgeries. Moreover, the session durations were extended for complex procedures, allowing participants adequate time to master advanced techniques. These refinements were guided by constructive feedback from the trainees and aimed at maximizing the educational impact of each session while addressing specific challenges identified in the earlier training cycles.

## Discussion

This study primarily aimed to evaluate the effectiveness of the cadaver surgical training program in enhancing surgical skills, confidence, and anatomical understanding among participating surgeons. We hypothesized that program completion significantly improves technical proficiency and safety, thereby serving as a highly beneficial off-the-job training platform applicable to real-world surgical practice. The current survey responses from participants indicated that 92% rated the training content as “good” or “excellent,” with all participants expressing satisfaction with the training experience. Moreover, a substantial majority reported that the skills acquired would be directly applicable to their clinical practice. These findings underscore the exceptional quality of the training environment and highlight its potential to instill confidence in trainees by mastering surgical techniques.

Traditionally, the surgeons predominantly rely on on-the-job training for surgical skills acquisition, where their techniques are developed through direct involvement in live surgeries. However, this approach is increasingly viewed as less favorable due to ethical concerns and patient safety considerations, leading to an increasing demand for off-the-job training allowing surgeons to refine their skills in simulated environments before performing procedures on patients. Training with live large animals provides a sense of realism and practical experience [12], but it is limited by anatomical differences between animals and humans, as well as ethical concerns regarding animal welfare [13]. Conversely, advancements in cadaver preservation techniques have enabled the use of specially embalmed cadavers, which closely mimic the anatomical and tissue characteristics of living bodies. Thus, cadaver-based surgical training has become a widely accepted and effective off-the-job training approach to enhance surgical proficiency in a realistic yet ethically sound setting [14, 15].

To date, we have conducted 13 cadaver surgical training sessions at CALNA, an on-campus facility that adheres to the guidelines for cadaver dissection in clinical medical education and research [9]. This program enables trainees to practice thoracic surgical techniques on human cadavers by using the same devices and instruments utilized in daily surgeries. However, standardized programs and accreditation for specialist certification are yet to be established in the field of thoracic surgery, partly due to the limited number of reports and documented outcomes. To address this gap, we developed a cadaver surgical training program that aimed to enhance the technical skills of young and mid-career thoracic surgeons, focusing on mastering fundamental and advanced surgical techniques.

In the current thoracic surgical practice, the use of minimally invasive approaches, such as VATS or robot-assisted thoracic surgery, has significantly increased, whereas the frequency of extensive surgeries requiring extended thoracotomies or specialized approaches has markedly declined [16]. Thus, the opportunities for young thoracic surgeons to acquire fundamental skills, such as open thoracotomy maneuvers, tactile assessment of surgical fields, comprehensive observation of the operative field, and dissection of pulmonary arteries or lobectomy through open approaches, have significantly diminished. Specifically, procedures involving less common approaches, such as the TMA and hemi-clamshell approach, are rarely encountered in clinical practice. Moreover, cadaver surgical training offers an invaluable opportunity to develop a deeper understanding of the bony thorax and intrathoracic anatomy, making it an ideal platform to address these educational gaps.

Our cadaver surgical training program is structured into three progressive levels: basic training, advanced training, and specialized techniques, including rare surgical approaches, VATS, and lung transplantation [8]. To refine the training further, the program’s effectiveness in enhancing surgical skills and its applicability to daily clinical practice should be quantitatively assessed. However, due to the variability in cadaver conditions affecting the difficulty and duration of the procedures, objective quantitative evaluation remains challenging. Consequently, this study used participant surveys for the numerical assessment. Based on the survey responses, 92% of the participants rated the training content as “good” or “excellent,” and the majority indicated that the skills acquired would be directly applicable to their clinical practice. As organizers, we strongly believe that it represents a meaningful and impactful training opportunity for thoracic surgeons.

This study has several limitations that should be acknowledged. First, one notable issue is the lack of mediastinal movement associated with cardiac pulsation and respiratory cycles in the current training setup. This limitation highlights the need for further advancements in surgical education research to enhance simulation realism. Second, the availability of cadavers is declining, posing a challenge to the sustainability of such programs. Addressing this issue will require increased institutional support, optimized resource management, and potential alternative training models. Third, the accessibility of cadaver surgical training remains limited, as it is currently restricted to academic institutions, making it less available to nonacademic centers. Expanding training opportunities through multi-institutional collaborations and standardized accreditation frameworks may help bridge this gap. Fourth, standardized and universally applicable training programs have yet to be established across multiple institutions. This underscores the need for collaborative efforts to develop a unified curriculum. Fifth, our current evaluation method lacks objective skill assessment, as training effectiveness has so far been assessed based on subjective impressions immediately after each session. To address this, we plan to implement the evaluation of participants' skills in a second cadaver session after the initial training. A structured approach, where the same trainees participate in paired sessions with the same instructor twice, would allow for a more scientific and objective assessment of training effectiveness. Finally, our study lacks follow-up data on how well the acquired skills translate into real-world surgical practice. To improve this, we plan to conduct follow-up surveys for past participants who have since performed surgeries that were simulated during the cadaver sessions. These approaches will provide a more objective measure of the program’s effectiveness.

In Japan, clinical experience of young surgeons remains limited, making it challenging for young surgeons to gain sufficient experience through on-the-job training alone. Therefore, training programs that optimize skill acquisition within limited timeframes and ensure direct applicability to daily clinical practice should be established [17].

In conclusion, we introduced a cadaver surgical training program at CALNA and evaluated its effectiveness by analyzing participant survey responses. Our findings showed that this training program significantly enhances surgical skills, instills confidence in performing procedures, and deepens participants’ understanding of the thoracic anatomy, highlighting the value of cadaver-based training in advancing thoracic surgical education. We aimed to further develop programs that enable novice surgeons to acquire advanced surgical skills within a short period. Therefore, achieving this objective will require leveraging our accumulated experience and fostering collaborative information sharing across multiple institutions to establish a standardized and effective training framework.

## Supplementary Information

Below is the link to the electronic supplementary material.

Supplemental Video 1 The Advanced Skills Course focuses on mastering less common and more complex thoracic surgical approaches and techniques, including the clamshell approach, hemi-clamshell approach, transmanubrial osteomuscular sparing approach (TMA), chest wall resection, pulmonary artery and bronchial reconstructions, and sleeve lobectomy. This movie shows a training session involving the TMA. The cadaver, preserved to closely mimic the texture and structural integrity of living tissues, provides a highly realistic training environment, including skin and bony thorax conditions. The process of sequentially identifying and dissecting the anatomical structures in the space between the clavicle and the first rib during TMA offers invaluable training. (MP4 19110 KB)

Supplemental Video 2 This movie shows a VATS training session. The procedure is performed by an eighth-year surgeon with no prior experience in VATS lobectomy. This cadaver had pleural adhesions, requiring the trainee to begin with adhesion dissection, closely simulating a real clinical scenario. The trainee opted to practice using a four-port, anterior-facing, inverted VATS technique and was performing a left upper lobectomy. After incising the anterior mediastinal pleura, the trainee proceeded with a vascular sheath incision and vessel dissection. Compared to training with large animals, one of the key advantages of cadaver surgical training is the ability to practice while confirming human anatomical structures. The session also included an assistant, providing an opportunity to practice camera handling and teamwork. Using electrocautery and energy devices, identical to those utilized in actual clinical practice, allows trainees to experience a highly realistic surgical environment. During the training, the trainee occasionally simulated the management of critical complications, such as vascular injuries by immediately performing maneuvers for compression and hemostasis. Such scenarios emphasize the importance of mastering techniques to avoid similar errors in actual surgeries. In this session, the trainee repeatedly practiced pulmonary artery dissection, making the most of the available training time. (MP4 27946 KB)

## Data Availability

Data supporting the findings of this study are available from the corresponding author upon reasonable request.
